# Systemic sclerosis with renal crisis and pericardial effusion

**DOI:** 10.1002/ccr3.3467

**Published:** 2020-11-02

**Authors:** Kalyan Prudhvi, Jayasree Jonnadula, Gurusaravanan Kutti Sridharan, Mary Dominguez

**Affiliations:** ^1^ Montefiore Medical Center Bronx NY USA; ^2^ Banner University Medical Center Tucson AZ USA

**Keywords:** cardiac tamponade, pericardial effusion, renal crisis, scleroderma, thrombotic microangiopathy

## Abstract

Scleroderma renal crisis occurs in 10% of patients with systemic sclerosis carrying a poor prognosis. A kidney biopsy is consistent with thrombotic microangiopathy (TMA) with clinical findings discerning it from other TMAs. Progression to ESRD occurs in 50% of patients which can lead to further complications necessitating emergent interventions including dialysis. Patients with scleroderma can have pericardial involvement with tamponade physiology requiring intervention such as pericardiocentesis.

## Graphical abstract

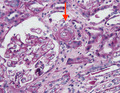

## 1 CASE

**FIGURE 1:**
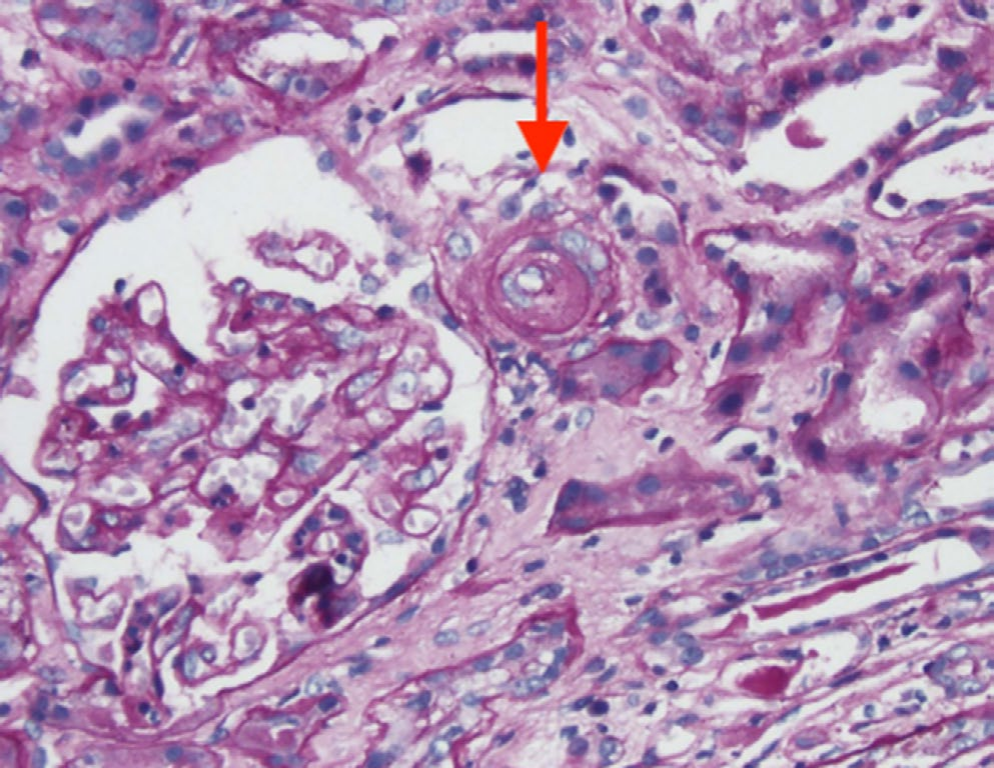
Kidney biopsy showing thrombotic microangiopathy (hematoxylin and eosin ×400). Collapsed capillary loops and mesangiolysis in glomeruli, arteriolar wall with intimal thickening with edema, mucoid change, and internal elastica duplication (red arrow)

A 70‐year‐old woman was hospitalized for elevated creatinine of 2.1 mg/dL dL and elevated blood pressures. Her urinalysis revealed 11‐20 RBCs and elevated urine protein‐creatinine ratio of 2.5 g/g. Autoimmune workup was positive for anti‐Scl‐70 antibody, negative for ANA, Ds DNA, citrulline antibody, rheumatoid factor, myeloperoxidase, and antiprotease 3. ADAMTS 13 activity was normal, and peripheral smear examination was negative for schistocytes. Her blood pressures were controlled with captopril 25 mg three times a day. Creatinine continued to uptrend from 2.1 mg/dL to 5.9 mg/dL. Her kidney biopsy revealed thrombotic microangiopathy. (Figure 1).

**FIGURE 2:**
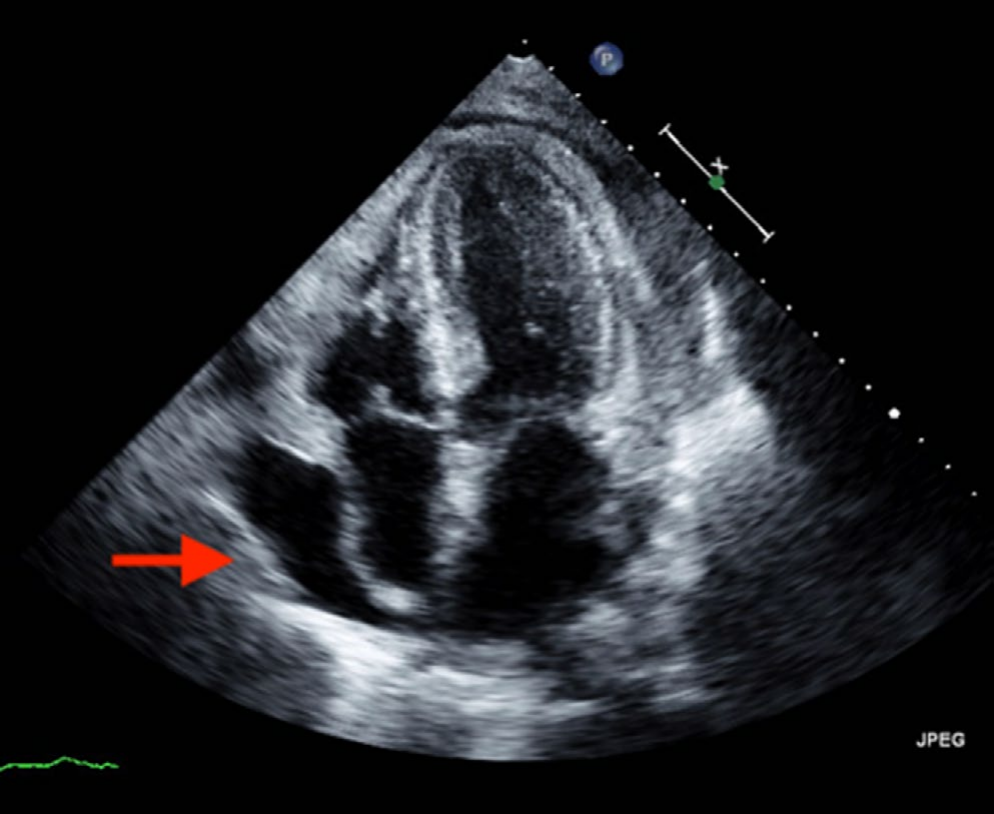
2D echocardiogram apical four‐chamber view. Late diastolic/ early systolic collapse of right atrium suggestive of tamponade physiology (red arrow)

Over the next 2 weeks, she had progressive dyspnea, chest pain, and bilateral lower extremity edema. Her serum blood urea nitrogen levels were elevated ranging from 90 mg/dL to 144 mg/dL. She was hypotensive and echocardiogram revealed pericardial effusion with tamponade physiology. (Figure 2) She underwent pericardiocentesis with pericardial drain placement and was initiated on dialysis and remained dialysis‐dependent.

Systemic sclerosis (SS) is a connective tissue disease involving multiple organ systems. SS is associated with TMA in 10% of cases and is referred to scleroderma renal crisis (SRC), which carries a terrible prognosis with mortality rate up to 30% ‐ 50% in 5 years.^1^ Blood pressure control with renin‐angiotensin‐aldosterone ( RAAS) blockade therapy is recommended. In our patient, pericardial effusion was attributed to Scleroderma or uremic pericarditis. Pericardial effusion with tamponade physiology is life‐threatening and requires emergent pericardiocentesis and dialysis initiation.^2^ 50% of patients with scleroderma renal crisis progress to ESRD. Renal recovery in scleroderma patients is 7%‐10%.^3^

## CONFLICT OF INTEREST

None.

## AUTHOR CONTRIBUTIONS

First and third authors: written the manuscript initial draft. Third author: worked on editing and finalizing the manuscript. Second author: worked on reviewer comments and resubmission.

## Data Availability

All data underlying the results are available as part of the article, and no additional source data are required.
